# Supplementary material for: Are carbon emissions trading and green financial instruments synergistic? -Comprehensive quantitative research based on content analysis
Source: PLoS One. 2024 Mar 7;19(3):e0298601. doi: 10.1371/journal.pone.0298601 (PMC10919601; doi:10.1371/journal.pone.0298601)
Supplement: S1 Data — (ZIP) [file pone.0298601.s001.zip › Data/地级市Z_F工作报告.zip!/╣π╢1⁄2/╖≡╔╜╩╨/└┤╫╘╣1⁄2╓┌║┼í╛┬φ┐╦╩2╛▌═°í┐_2011.docx]

各位代表：

现在，我代表佛山市人民政府向大会报告政府工作，请予审议，并请政协各位委员和其他列席人员提出意见。

五年奋进 实现大跨越

“十一五”时期，是佛山全面贯彻落实科学发展观，战胜国际金融危机，推动产业转型、城市转型和环境再造取得重大突破的五年;是全市人民团结奋进、务实创新，胜利完成“十一五”规划主要目标和任务，经济社会发展取得重大成就，更多改革开放成果普惠民生的五年。在这五年里，我们：

——应对危机率先突围。早谋快断，赢得主动，有效应对金融危机冲击，实现经济率先突围。危机中，全市人民精诚团结，政企同舟共济，化“危”为“机”，赢得了社会各界的赞誉。

——经济实力显著增强。地区生产总值、工业总产值、财政收入与“十五”期末相比，全部实现翻番。2010年，地区生产总值达到5651亿元，五年连跨3个千亿元大关；来源于佛山的财政总收入1039.96亿元，其中地方一般预算收入305.96亿元。

——发展质量优化提升。经济增速与经济效益同步提高。2010年，纳税超千万元以上企业729户，是2005年的2.39倍；五年新增上市企业16家，超过以往时期总和；新增中国驰名商标37件、中国名牌产品25个，参与研制国家及行业标准457个。

——人民生活持续改善。2010年，城镇居民可支配收入27245元、农村居民纯收入12202元，分别比2005年增长56.4%和61.1%；金融机构本外币存款余额达到8462亿元，其中城乡居民储蓄存款4461亿元，分别比2005年增长116.6%和80.9%。

闪光的数字背后蕴含了无数人的心血，收获成绩的同时也收获了精神。

一、以佛山人精神凝聚发展共识

大力弘扬“敢为人先、崇文务实、通济和谐”的佛山人精神，使之成为佛山人和谐共进、勇攀高峰的信念。

敢为人先，体制创新迸发新活力。深入学习实践科学发展观，先行先试，力推体制机制改革。率先实施区大部制改革、镇（街）简政强镇事权改革，行政效率、服务效能得到提升。深化行政审批制度改革，共向区下放审批事权493项。统筹城乡发展、金融发展三项计划等改革全面展开。通过改革，全市人民发展热情高涨，改革开放初期那种敢闯敢试、创新进取的精神，又一次汇聚成为推动佛山又好又快发展的强大动力。

崇文务实，统筹协调赢得新机遇。以“一环”全线开通为契机，推动全市五区交通路网衔接，解决城市间分割状态；通过“五区同创，城乡共创”国家环保模范城市活动，密切区域间交流合作；通过岭南文化的情感纽带，增进城市间凝聚力。正是这种内部连接，优化了城市发展格局，促进了全市五区融合。把握《规划纲要》实施机遇，主动与广州全方位对接，接受广州科技、人才、信息等辐射带动。广佛地铁顺利开通，佛山由此进入地铁时代，城市发展模式和人民生活方式正在更新。通过佛港澳合作，佛山拥有一个向世界推介自己的全新平台，经济现代化和城市国际化步伐不断加快，发展基础更加扎实，发展信心更加充足！

通济和谐，开放兼容展现新面貌。“以和为贵”的特质使佛山人勇于应对各种困难和挑战。面对金融危机冲击、亚运安保、环保、社会稳定等问题，全市上下一心，积极寻求破解之策，促进了经济社会平稳较快发展，形成了政府引领能力增强、企业家素质提升、市民文明素养提高、政通人和的生动局面。佛山人视野更开阔，胸襟更宽广，融合精神更彰显，逐步走出了小佛山概念，认识到固步自封没有出路，携手共建方能共赢，这是佛山人收获的宝贵精神财富！

二、以科学发展观引领转型再造

准确把握国际国内发展变化新趋势，坚持科学发展，优化提升发展质量和效益，力促产业转型、城市转型和环境再造。

产业转型成效明显。确立“精细发展农业，优化发展工业，加快发展服务业”战略，推动产业与科技、金融相融合，促进产业结构优化升级。农业产业化现代化水平不断提高，五年建设现代农业园区26个，新增农业龙头企业33家。实施“三个一批”和“双转移”战略，调整优化工业结构，五年关停高耗能、高污染企业600多家。传统产业生机勃发，新兴产业迅速崛起，现代服务业亮点纷呈。资本之光照耀产业高地，五年政府出资引导民间资本约20亿元进入创投行业，运用财政杠杆撬动担保贷款约345亿元，缓解了中小企业融资难问题。广东金融高新区成为现代金融产业后援服务基地，累计引进39家知名机构和项目。科技创新助推产业转型，五年投入更新改造资金1143亿元,是“十五”时期的4.3倍。全面展开与中科院的战略合作，共建产业技术创新与育成中心。内外需协调拉动经济增长，五年全社会固定资产投资6448亿元、社会消费品零售总额5780亿元、出口总额1339亿美元，分别是“十五”时期的2.81倍、2.34倍和2.42倍。内外源型经济“双轮驱动”，2010年民营经济增加值达3419亿元，比2005年增长116.4%；五年吸引外商直接投资83.1亿美元，是“十五”时期的1.73倍，新增世界500强项目25个。

城市转型步伐加快。以基础设施建设和“三旧”改造为抓手，优化城市形态，重塑佛山新貌。基本形成现代立体交通格局，五年新增高速公路303公里，路网通行能力有效提高，市域内外人流、物流进一步畅通。实施公交优先战略，推行公交共同体模式改革，公交发展水平明显提高，服务质量持续改善。东平新城规划建设取得实质性进展，一批重点项目和基础设施建设全面展开。率先实施“三旧”改造，创造出“佛山模式”。

环境再造成绩突出。大刀阔斧推进环境整治，城市环境质量持续好转，走出了一条人与自然和谐、城市与产业相容的新路子，创建成为国家环保模范城市。以大气和水环境治理为重点，倒逼机制与退出机制并用，工程减排、结构减排和监管减排多措并举，推进重点行业、重点区域环境污染整治，提前完成“十一五”环保目标任务。五年新增污水处理能力175.5万吨/日，2010年城镇生活污水处理率达到77%，汾江河基本实现“江水变清，不黑不臭”的阶段性目标。实施“三年促变，绿地佛山”战略，五年新增和改造绿地面积4762万平方米，城市整体绿量明显增加，区域（省立）绿道全线贯通。

三、以人为本促进社会和谐

牢固树立发展为民的思想，加大财政保障力度，用情用力保民生，尽心尽责筑和谐，营造了安定有序的发展环境。

民生改善更加有力。五年投入民生事业702亿元，占全市一般预算支出的56%。办成、办好、办妥系列惠民工程和一批民生实事。城乡九年义务教育全面免费，教育现代化和教育综合改革走在全省前列。建立健全养老保险、社会救助及住房保障体系，五年解决5836户低收入家庭住房困难。率先启动实施“新农保”制度。基本形成覆盖城乡的公共卫生网络，建立住院、门诊全方位医疗保障体系，在全省率先进入全民医保时代。就业形势稳定，五年新增就业人数97.4万人，城镇登记失业率控制在2%以内。加强文化基础设施建设，打造“魅力佛山”系列文化品牌，祖庙完成百年大修。成功举办12届省运会，承办亚运会佛山赛区赛事。稳定低生育水平，在全省率先实行婚前、产前免费检查。汶川水磨重建任务圆满完成，扶贫开发工作进展顺利。

社会大局和谐稳定。推动警力“下沉”，实施科技强警，社会治安防控体系进一步健全。开展系列严打整治，社会秩序明显好转。完善社会矛盾调处机制，设立领导大接访制度，建立镇（街）综治信访维稳中心，及时解决群众反映的实际问题。加强应急管理工作，成功应对各种突发事件。开展产品质量、农产品、食品、药品安全整治，强化安全生产监管，公共安全水平不断提高。军政军民团结进一步巩固，6次荣获“全国双拥模范城”称号。

民主法制建设稳步推进。五年共办理人大代表建议和政协委员提案1273件，办复率达到100%。全面加强依法治市建设，完成“五五”普法任务。深入推进政府信息公开，全市村（居）全面引入“第三方”管理农村财务，实现规范透明理财。组建市政府专家顾问团，实行重大事项公众参与、专家论证制度，促进民主科学决策。认真落实党风廉政建设责任制，深入开展机关作风建设系列活动，运用现代信息技术手段加强行政监察，严肃查处违纪违法案件，政风行风明显好转。

各位代表！五年奋斗，成绩显著，这是省委、省政府和市委正确领导的结果，是全市人民齐心协力、拼搏进取的结果，是市人大和市政协监督支持的结果。在此，我代表佛山市人民政府向全体市民，外来务工人员，中央、省驻佛山单位，驻佛山人民解放军指战员、武警官兵和社会各界人士表示崇高的敬意，向长期关心支持佛山建设的港澳台同胞、海外侨胞、国内外友人表示衷心的感谢！

在看到成绩的同时,我们也清醒地认识到，在前进的道路上还面临不少困难和问题，主要是：生产要素约束凸显，经济结构仍需改善，科技创新能力不够强；城市建设和管理相对滞后，统筹城乡发展有待增强；社会法治意识相对薄弱，和谐佛山建设还有不少隐忧。对于这些问题，我们一定高度重视，在“十二五”时期采取有力措施认真加以解决。

继往开来 谱写新篇章

未来五年，是实施“十二五”规划、加快转变经济发展方式、全面推动佛山转入科学发展轨道的黄金时期，是落实“四化融合，智慧佛山”战略，阔步迈进民富市强幸福佛山的关键时期。

未来五年，我们将全面贯彻落实中央、省和市委的各项战略部署，以科学发展为主题，以加快转变经济发展方式为主线，以“民富市强，幸福佛山”为核心，以“四化融合，智慧佛山”为路径，突出统筹兼顾，突出科技进步，突出改革创新，加快建设现代制造基地、产业服务中心、岭南文化名城、美丽幸福家园，把佛山建设成为一座充满朝气、人与自然和谐相处的全国文明城市。

“十二五”时期的主要奋斗目标是：地区生产总值年均增长10%，力争“十二五”期末超万亿元；人均生产总值超2万美元；城乡居民收入较快增长，力争与经济增长同步，城乡收入差距有所缩小；城镇登记失业率控制在3.5%以内；人口自然增长率控制在7.73‰；万元GDP能耗完成省下达指标。

今年是实施“十二五”规划的开局之年。今年主要经济指标预期是：地区生产总值增长12%；人均生产总值增长11%；单位GDP能耗下降、主要污染物减排量均完成省下达任务；地方财政一般预算收入同口径增长10%；居民消费价格总水平涨幅控制在4%左右。

为实现上述奋斗目标，我们必须做到：

一、转变经济发展方式，构建现代产业体系

目前我市每平方公里土地产出率为1.48亿元，与先进国家和地区相比，未来发展潜力巨大，必须有信心。根据我市特点，高产出主要来自于高水平产业，尤其是以制造业为主的现代产业。

1．探索农业发展新模式。用工业园区的集约理念发展农业，用城市规划的举措改造农村，用市场经济的规则和法治精神提高农民文明素质。加强农田保护，大规模建设现代标准农田，规划建设一批农业生态园区。加快发展设施农业和农产品物流业，积极推广农业产业化新模式，扶强扶大农业龙头企业和农民专业合作社，加大科技和金融支农力度，促进传统农业向都市型、生态型、观光型和外汇型农业转型发展。

2．力促制造业领跑广东。实施传统制造业改造提升、新兴产业培植发展“双轮驱动”，力争2015年工业总产值达到3万亿元，成为全省最大的制造业城市。推进国家信息化与工业化融合试验区建设，加快运用信息技术、先进适用技术改造提升家用电器、陶瓷建材、纺织服装、有色金属及制品等优势传统产业，实现传统产业现代制造，重点开发智能家电、智能家居等智能产业和产品。用好国家新型工业化产业示范基地政策，着力引进光电、新材料、生物医药、环保、新能源汽车等新兴产业龙头项目，发展壮大汽车及零配件、机械装备等先进制造业，重点支持南海经济开发区，乐平、沧江工业园等建设，全力打造白色家电、新型显示器件、现代陶瓷三大世界级和九大国家级产业基地。

3．力推第三产业加速提效。坚持服务业与制造业相融合、相促进，重点发展金融业、现代物流、工业设计、科技信息、总部经济等生产性服务业，鼓励引进律师、会计、评估等专业服务业，合理布局生活性服务业，完善发展服务业的政策措施，建成一批现代服务业集聚区。探索制造业主辅分离，选择产值在亿元以上的若干企业试点，促进企业走专业化、集聚化道路，提升第三产业发展水平，力争“十二五”时期第三产业增加值年均增长15%。

4．大力招商引资。招商引资是增强经济发展后劲的重大利器，任何时候不能松懈。树立“大招商、招大商”理念，把握后金融危机时代国际产业转移的新趋势、国内大企业布局华南地区的新机遇，创新招商引资方式，注重内生增长与嫁接增长、产学研带动与金融带动，针对战略性新兴产业、先进制造业和现代服务业，集中力量引进一批龙头大项目，促进国内外大企业落户佛山，力争“十二五”时期新增世界500强企业20家，利用外资总额达到120亿美元。

5．深入扩大内需。积极促进消费结构升级，大力培育文化娱乐、体育健身、旅游休闲等消费热点。引导企业大力开拓内地市场和农村市场，扩大佛山产品国内市场份额。力争“十二五”时期全社会固定资产投资年均增长12%；2015年，全社会消费品零售总额超3000亿元，外贸出口总额超500亿美元。

6．强化金融、科技、人才支撑作用。促进金融、科技、人才与产业相融合，让金融、科技成为产业发展的“双翼”，让人才成为产业发展的助推器。大胆创新金融发展，引进培育各类新型金融机构，“十二五”时期，辖区内5家农信社全部改制为农商行，至少组建5家村镇银行，小额贷款公司、担保公司分别达到20家和80家以上，创投基金超过50支。鼓励民间资本参与金融业发展，促进民间资本与产业有效融合，带动佛山民间财富增长。积极推进广东金融高新区建设，支持企业成为“新三板”试点，大力推动企业上市，力争2015年上市公司超过80家，打造证券市场“佛山板块”。发挥财政导向作用，引导各类金融机构支持科技研发，形成多元化科技投入体系，力争2015年全市研究开发经费占GDP比重达到2.3%。进一步密切与中科院、国内外高校的合作，力争每年引进一所知名科研院所与佛山共建产学研合作平台。支持“中国南方智谷”建设，打造一批国家级、省级创新平台，推动核心技术、共性技术和关键技术的创新和转化。重点建设佛山高新区创新创业园，发展一批科技孵化器项目，让一批创新成果实现产业化。在全社会营造尊重人才的良好氛围，让企业家懂得投资人才比投资设备往往更有效，引导企业重视人才、善待人才，从分享“人口红利”到分享“人才红利”。完善人才落户、住房、税收等优惠政策，建立“人才绿色通道”，让千军万马扎根佛山。以产业和项目为载体，大力引进产业领军人才和科技创新团队，2015年高层次人才总量达到2.5万人。

7．破解土地、环境容量瓶颈。土地和环境容量是未来我市发展的两大制约因素，必须找到抓手予以突破。以“三旧”改造破解土地瓶颈。今年市政府已出台1号文件，明确要求禅城区不低于40%、其他区不低于60%的旧厂房、旧物业改造面积，优先用于发展工业和现代服务业。从今年起，连续三年市、区财政每年共拿出10亿元，进行土地储备和鼓励旧厂房、旧物业的土地流动，推动连片开发。以节能减排提升环境容量。严格环保准入，通过调整产业结构、优化能源结构、运用科技手段等方式促进减排，实现经济与环境相融共赢。组织家具、纺织、铝型材等重点行业专业平台，集中精力研究开发行业环保技术，推动绿色制造、低碳发展。建立污染减排长效机制，严厉打击各种环境违法行为，坚决不能以牺牲环境求发展！

二、建设和谐宜居城市，全面提升城市品质

城市能让生活更美好，也可能降低生活质量，在地区竞争中城市建设与管理殊显重要，我们必须以前所未有的高度加以认识。

8．打造“佛山新城区”。实行“强中心”战略，加速推进东平新城开发建设，力争通过几年的努力，将其打造成为广东工业服务示范区、具有现代岭南特色的中心城区。坚持“统一规划，分类实施”方针，把东平新城总规划面积划分为核心区、重点控制区、协调区三个层次分类推进、市区联动，促使佛山新城区尽快成型，展现英姿。发挥佛山新城区引领带动作用，联接、促进禅城-桂城、大良-容桂两大组团互动发展，形成佛山城市中轴线，彰显现代化大城市风貌。

9．深化组团城市建设。坚持组团城市发展道路，强化全市统筹和引导，推动规划实施评价考核，确保规划有效实施。支持各组团在全市统一规划框架下，依据自身功能定位、产业布局和公共服务，进行差异化的开发建设，形成“1+2+5”组团城市发展新格局。进一步健全组团城市建设管理体制和跨部门协调机制，加速推进组团城市交通、通信、供电、供气、给排水等基础设施一体化和智能化。以畅通人流、物流为目标，深化组团之间快速公路、轨道、快线公交等建设，打通组团之间交通瓶颈；调整优化现有交通路网格局，力争将过境国道迁至区域边界。加快佛山西站、东平新城交通枢纽中心、佛山引航中心、港口、公交站场等综合交通枢纽建设，促进各类交通运输方式“零换乘”和无缝对接。建设智能交通信息系统，完善公共自行车服务网络，初步形成智能交通服务体系。优化公交布局，完善市、区、镇、村四级公共交通网络，改善公交服务水平，逐年提高公交分担率，力争2015年达到35%。以“三网融合”为抓手，加快推进互联网、物联网和云计算等信息技术在城市建设与管理各领域的应用，构建全市统一的智能服务基础平台，打造智能城管、数字城管。充分利用网络、微博等完善管理薄弱环节，发动市民行动起来献言献策，全面提升城市综合管理、科学管理、精细管理水平，做到智能高效、秩序井然。

10．推动广佛同城化发展。全面落实《广佛同城化发展规划》，创新同城化发展的体制机制，进一步提高同城合作效率，率先在产业、交通、通讯、金融、社会事业、公共服务等领域实现同城，2015年基本实现广佛同城化，使广佛都市圈成为珠三角强有力的新经济增长极，示范携领珠三角一体化建设。

11．加快城乡一体化发展。深化统筹城乡综合改革试点工作，实施农村居民收入增长计划，提高农村居民文化素质。建设一批名镇、名村，示范带动宜居城乡发展。深化农村体制改革，完善农村股权分配和流转机制，稳步推进“村改居”工作，改革基层组织管理体制和实施政经分离。建立农村集体资产管理交易平台和农村财务监控系统，以现代信息技术加强农村管理。

12．创建全国生态文明城市。用“大环保”理念和科技信息手段，深入推进水、大气和环境卫生综合整治。实施污染物排放总量控制，重点整治小锅炉、挥发性有机污染物和机动车尾气排放，五年内全部淘汰黄标车和小锅炉。强化内河涌治理，加强饮用水源水质保护，提高固体废物污染防治水平，2015年，城镇生活污水处理率达到85%，城镇生活垃圾无害化处理率达到90%，土壤污染得到有效防治。完善防洪减灾工程体系，提高水患应对能力，让水真正成为我们的财富和朋友。以创建全国绿化模范城市为目标，深入开展植树造林活动，完善城市道路绿网和水系绿网骨架，增加中心城区绿量，规划建设一批湿地公园、滨江公园、农业生态公园等绿色开敞空间，发展一批“绿色居住区”，显著提升城市生活质量。2015年人均公园绿地面积达到11平方米，成为蓝天碧水、绿意盎然的花园城市。

三、统筹发展民生社会事业，建设民富市强幸福佛山

坚持发展为了人民、发展成果由人民共享，使佛山市民学有优教、劳有多得、病有良医、老有善养、住有宜居，生活更幸福。

13．打造智能教育。民富根本在于教育。打造“佛山e教育”品牌，推动信息化与教育相融合，建立城乡一体的社区教育网络和数字教育公共服务平台，促进学习型佛山建设，使全体市民享受终身教育。开展教育综合改革试点工作，全面提升学前教育质量，促进各类教育均衡协调发展，2015年在全省率先基本实现教育现代化。

14．发展智能医疗。健康是民生之本。加快“健康e园”工程建设，构建基于市民电子健康档案的佛山区域卫生信息平台，让人人拥有一个电子化的家庭医生，2015年全市居民电子诊疗和电子基本公共卫生服务可及率达到90%以上。加大医药卫生体制改革力度，强化基层医疗卫生机构建设，构建城乡一体的医疗服务和公共卫生服务体系。积极推进公立医院改革，鼓励社会资本进入医疗卫生领域，满足多样化的医疗卫生需求。全面推行基本药物制度，逐步解决市民“看病难、看病贵”问题。大力推进中医药进社区，建设中医药强市。加强人口计生工作，提高人口素质。

15．建设智能文化。文化是一座城市的灵魂。坚持文化与产业对接、与城市结合、与人相融，让文化促进产业发展、城市提升、民生改善。加快智能图书馆等公共文化设施建设，传承发展陶瓷文化、粤剧文化、武术文化等岭南文化神韵，打造西樵山等一批旅游文化品牌，建设一批大型城市文化街区和文化新地标，塑造高品质的城市文化形象。

16．创建全国文明城市。举全市之力，形成五区联创、城乡联创、全民共创格局。对照要求，查找不足，实施重点突破、难点攻关，组织开展交通秩序、市容市貌、社会治安、集贸市场、窗口服务等专项整治，确保城市综合环境全面改善，使社会环境、城市管理、人文素质上一个新台阶。

17．提升市民幸福感。扎实办好一批民生实事，使广大市民共享发展的“幸福成果”。着力统筹城乡就业服务，重点解决高校毕业生、农村转移劳动力、城镇就业困难人员的就业问题，倡导勤劳创业，防止养懒罚勤。持续扩大养老、医疗等五大险种参保人数，不断提高待遇水平，实现“新农保”全覆盖。加快完善住房保障体系，大力建设公共租赁房，切实解决中低收入家庭的住房困难。财政要雪中送炭，重点对弱势困难群众提供教育、医疗、住房、低保等救助。发展居家养老服务，支持残疾人事业。切实保障外来务工人员合法权益，使他们切实感受到党和政府的温暖。继续做好对口援建和扶贫开发工作，力争用两年时间，扶持高明革命老区贫困户基本实现稳定脱贫。健全社会治安防控体系，强化公共安全应急处置，严格落实安全生产责任制，加强农产品、食品、药品质量监管，让生活在佛山的市民更有安全感！

“十二五”主要指标与战略目标明确后，核心在落实，早抓早主动，迟抓更被动。必须将上述各项指标分解到每年、每月、每日的具体工作中，尤其要分解到各区、镇（街）、各部门，以及每一个人。2011年是“十二五”开局之年，不能简单以每年五分之一来对待，而是要力争开好局。政府将探索建立每季度工作考核制度，做到人人有责任、事事有落实、件件有结果。

各位代表！做好上述工作，必须加强两项建设：

一是加强法治社会建设。发展与否看稳定，稳定与否看法治。建设法治文明模范城市，是我市“十二五”重要任务。政府要成为法治建设的先行者，坚持依法行政。加强宣传教育和舆论引导，推动形成依靠法律、尊重法律的社会潮流。全体市民平等接受法律的约束和保护，征地拆迁、劳资纠纷、农村分红等信访问题均要导入法律程序来解决，让法律说了算。维护司法公平正义，确保程序公开透明，确保诉求依法办理，弘扬社会浩然正气。始终坚定一个共识，支持民主和法治，反对专权和腐败，推动佛山率先走进“法治文明”。

二是加强政府自身建设。继续深化区级大部制和镇（街）简政强镇事权改革，合理整合有关部门职能，建立协调有力、运转高效、监管到位的权力运行机制。科学划分市区事权，该放的放活，该收的收好，突出城乡规划、组团布局和重大项目建设全市统筹。根据各区发展的差异性，改进考核体系和配套政策，促进五区竞合发展。建立重大工作任务倒逼机制，激发公务员干事创业的智慧和干劲。加强公务员队伍建设，尤其要活化市直公务员队伍，使之既能够“走上去”，争取上级的支持；又能够“走出去”，善于抓项目、引外资；还能够“走下去”，善于统筹、指导和帮助各区解决实际问题，成为基层遮风挡雨的伞。根据业绩选人用人，实施市级机关绩效考评。强化信息化应用，精简文风会风，力争成为珠三角最高效、开明的政府之一。加强政府廉政建设，自觉接受人大监督，充分依靠政协智慧，努力建设一个清正廉洁、务实高效、人民信赖的政府！

各位代表！美好的蓝图正等待我们去描绘，人生的意义就在积极奋斗中。让我们在市委的领导下，立即行动起来，大干一场，建设民富市强幸福佛山！
